# Sclerotherapy and prolotherapy for chronic patellar tendinopathies - a promising therapy with limited available evidence, a systematic review

**DOI:** 10.1186/s40634-020-00303-0

**Published:** 2020-11-09

**Authors:** Oliver Morath, Manuel Beck, Jan Taeymans, Anja Hirschmüller

**Affiliations:** 1 Institute of Exercise and Occupational Medicine, Department of Medicine, Faculty of Medicince, Medical Center-University of Freiburg, Hugstetter Str. 55, D-79106 Freiburg im Breisgau, Germany; 2 Clinic for Orthopaedics and Traumatology, Department of Surgery, Faculty of Medicine, Medical Center-University of Freiburg, Hugstetter Str. 55, D-79106 Freiburg im Breisgau, Germany; 3 Bern University of Applied Sciences – Health, Murtenstrasse 10, CH-3008 Berne, Switzerland

**Keywords:** Patellar tendon, Sclerotherapy, Prolotherapy, Tendinopathy, Injections, therapy

## Abstract

**Abstract:**

Chronic Patellar tendinopathy (CPT) is a frequent overuse disorder in athletes and active people. Sclerotherapy (ST) and prolotherapy (PT) are, among a wide range of conservative treatment options, two promising therapies and have shown positive results in other tendinopathies. Since the treatments’ efficacy and safety are still not defined, this review sought to answer questions on recommendations for use in clinical utility, safety, and how to perform the injection in the most effective way. An electronic database search was conducted following the PRISMA guidelines. Inclusion criteria were set up according to the PICOS-scheme. Included were athletes and non-athletes of all ages with diagnosed painful CPT. Studies including patients suffering from patellar tendinopathy which can be originated to any systemic condition affecting the musculoskeletal system (e.g. disorders associated with rheumatism) and animal studies were excluded. Methodological quality (modified Coleman Methodology Score) and risk of bias (Cochrane Risk of Bias Assessment Tool 2.0) were assessed by two independent reviewers, with disagreements resolved with a third reviewer. The search yielded a total of 416 entries. After screening titles, abstracts, and full texts, ten articles were found for qualitative analysis. The mean Coleman Score was 64.57. Three randomized-controlled trials showed positive results with an increase in VISA-P score or a decrease in VAS or NPPS, respectively. The non-randomized studies confirmed the positive results as well. Among all ten studies no serious adverse events were reported. Based on this limited set of studies, there seems to be some evidence that ST and PT may be effective treatment options to treat pain and to improve function in patients with CPT. To strengthen this recommendation, more research is needed with larger volume studies and randomized controlled studies with long term follow up.

**Level of evidence:**

**IV**

**Supplementary Information:**

**Supplementary information** accompanies this paper at 10.1186/s40634-020-00303-0.

## Introduction

Tendinopathy is known as an overuse condition that often occurs in athletes or physically active people. Its cardinal symptoms are defined as pain, swelling and impaired function [43]. Besides Achilles tendinopathy (AT), chronic painful patellar tendinopathy (CPT), also referred to as jumper’s knee, is a frequently seen disorder. Its overall prevalence among elite athletes is estimated at 14.2%, with the highest incidence in volleyball (44.6%) and basketball (31.9%) players [41]. Eccentric training has shown the best evidence for treating CPT, but the less time consuming and most efficient treatment option is still under debate [19, 57]. Conservative treatment modalities include eccentric training, extracorporeal shockwave therapy, or physiotherapy. Research also reviewed the role of two injection therapies such as Sclerotherapy (ST) and Prolotherapy (PT) [15, 19]. After discovering that the origin of pain in tendinopathy arises from nerve endings runs parallel to the small vessels into the tendon, Alfredson et al. assumed that sclerosing the neovessels would destroy the nerve endings, resulting in pain relief [4–6]. Sclerotherapy was initially used for treating varicose veins [58]. The most commonly used substance is Polidocanol, a topical anaesthetic, causing endothelial damage by interacting with the lipid layer of intimate cells’ membranes [18]. Other detergents that can be used for ST are sodium tetradecyl sulfate and sodium morrhuate [18]. In 2002 Ohberg and Alfredson conducted a pilot study investigating the effect of ST on chronic painful Achilles tendinopathy and showed encouraging results [49]. Further research may confirm these findings [6, 68]. However, there are reports that do not support that the neovessels are the source of pain [17, 63].

In the 1950s it was George Hackett who first introduced Prolotherapy [24].(PT). The motive behind PT is similar to ST. The mechanism of action of the mostly used substances, eg. hypertonic dextrose solution, is mainly generated by osmotic shock and dehydrating cells [7, 69]. By causing local damage to the tendon it is assumed to induce several effects including an inflammatory response which results in a healing process. The detailed mechanism remains unclear but seems to be multifactorial. Sensorineural analgesic pathways are discussed as well [51, 52]. In pilot studies, Lyftogt and Maxwell also yielded promising results for PT when treating the Achilles tendon in patients with AT. Lyftogt injected hyperosmolar dextrose around the paratenon and Maxwell targeted the tendon [42, 44].

Recently a systematic review with meta-analysis suggested that there is weak evidence that ST and PT might be effective treatments to treat pain in patients with AT [46]. As there have been promising results on the treatment of painful AT, this present systematic review is aimed at evaluating the effect of ST and PT on chronic painful patellar tendinopathy (CPT). By systematically analysing the available literature this review sought to answer questions on recommendations for the clinical use, safety of the procedure, and how the injection is performed effectively. Considering that this review’s aim was to make a recommendation on the clinical use in humans, animal studies were excluded.

## Material and methods

This systematic review was conducted following the PRISMA guidelines [45]*.*

**Fig. 1:**
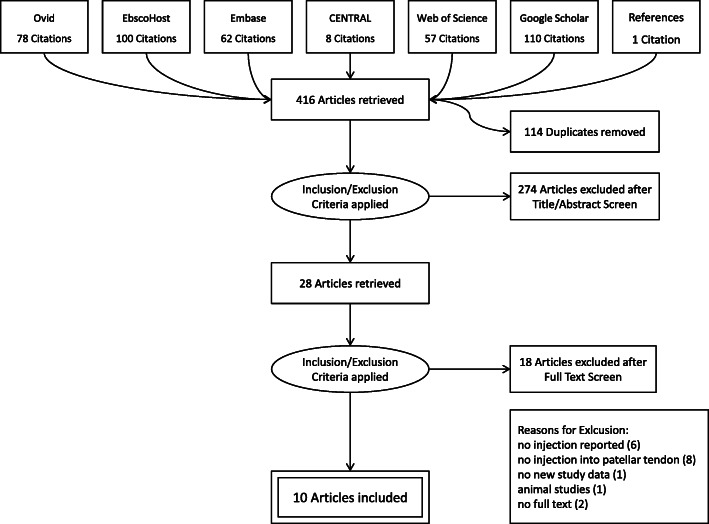
Flowchart of the selection process according to PRISMA guidelines

The flow-chart (Fig. 1) depicts the different steps of the review procedure.

### Study protocol

A study protocol has been edited and registered in 20/02/2019 on PROSPERO- the international prospective register of systematic reviews. The PROSPERO registration number is *CRD42019125803*. The protocol can be accessed via https://www.crd.york.ac.uk/prospero/display_record.php?RecordID=125803 or can be provided on request.

### Eligibility criteria

Eligibility criteria for suitable articles were set up according to the PICOS scheme (Population, Intervention, Outcome and Study design). Included were athletes and non-athletes of all ages with diagnosed painful CPT. Studies including patients suffering from patellar tendinopathy which can be associated with any systemic condition affecting the musculoskeletal system (e.g. disorders associated with rheumatism) and animal studies were excluded. Studies on interventions with injection treatments that describe a sclerosing mechanism of action addressed to destroy neovascularisations or prolotherapy (e.g. hyperosmolar glucose solution) were included. The injection had to target the patellar tendon. Control groups included patients that received any other treatment, such as surgery (e.g. arthroscopic shaving), other injection treatment (e.g. platelet-enriched-plasma, glucocorticoids), conservative treatment (e.g. eccentric training), different dosages or placebo. Studies investigating pain and function as outcome variables were included in this review. All published studies describing one of the following designs were eligible for further analysis: randomized-controlled trial (RCT), quasi RCT, prospective study, retrospective study, and case reports. There were no restrictions in language and date of publication.

### Search

The following electronic databases were searched: MEDLINE via Ovid, BIOSIS Previews and Archive via Ovid, CINAHL via EBSCOHost, SportDiscus via EBSCOHost, CENTRAL (Cochrane Central Trial Register of Controlled Trials) refined to trials via Cochrane Library, Web of Science, Embase. The last database searches were conducted on 27 February 2019 and on 8 March 2019 (Embase) respectively. To find additional topic-related publications, a Google Scholar search using the term “patellar tendinopathy sclerotherapy prolotherapy” was performed. Furthermore, the reference lists of the eligible articles were screened for further yields. The search strategy was based on the search strategy of a previous work from the same group and adjusted according to the new topic [46]. The search strategy was developed by two reviewers (OM, AH). The full Ovid search strategy is stated in the Additional file 1.

### Study selection

After removing all duplicates, titles and abstracts of the studies were screened by two reviewers independently (OM, MB) by the help of “Rayyan” a free web-based semi-automatic screening tool [50]. Disagreements were discussed and resolved by seeking consensus and by the help of a third independent reviewer (AH). When title and abstract met inclusion criteria full text was assessed for eligibility. Again, disagreements were resolved by the help of a third reviewer (AH).

### Data collection

As the primary outcome measure, level of pain by VAS (visual analogue scale), level of dysfunction by the VISA-P score (Victorian Institute of Sport Assessment -Patellar score) or any similar method for measuring was defined. Visual Analogue Scale is a single-item tool to quantify the level of pain on a 0–100 mm linear scale (0 the best, 100 the worst). VAS score has shown good reliability to assess disability in patients with chronic musculoskeletal pain [10]. The VISA-P score is a multi-item questionnaire measuring pain and dysfunction of the patellar tendon. It is a reliable instrument to judge the clinical severity of patellar tendinopathy [65]. Further outcome measures could be the tendon thickness measured by ultrasound control, the safety of the intervention measured by the number of reported adverse events (AE), patient’s satisfaction with the treatment, as well as the time to return to the same level of sport respectively to previous level of activity and the usage of different substances or dosages.

Individual study data were extracted and imputed manually into a spreadsheet using the full text by OM and confirmed by AH. Author names, year of publication and the study type were extracted (study details). Number of patients (split in male and female), the number of treated tendons, the mean age of the participants, the mean duration of symptoms and mean follow-up were also extracted (population details). Three-month follow-up (12 weeks) was defined as main follow-up for data extraction. Furthermore, data on substance and dosage, control groups, pre and post outcome values from primary parameters (i.e. VISA-P or VAS score) and AE were extracted (intervention data). Central tendencies, measures of variability and *p*-Values of the outcome parameters under investigation were extracted (outcome details).

In case of incomplete or missing data, corresponding authors were contacted. This was unsuccessful for three studies [31, 61, 67]. Nevertheless, these studies were included and only the presented data processed.

### Quality and risk of bias

The risk of bias was double-assessed by two reviewers independently (OM, MB) by using the Cochrane Risk of Bias Assessment Tool 2.0 to evaluate the RCTs [28]. We did not apply the Cochrane Risk of Bias Assessment Tool for the non-RCTs, since it was specifically designed for the evaluation of RCTs and is not easily applicable for the non-RCTs [29]. The methodological quality of the studies was independently analysed by two reviewers (OM, MB) using the modified Coleman Methodology Score (mCMS) [34]. Disagreement was minor, discussed and resolved by seeking consensus by the help of a third independent reviewer (AH). Classification and interpretation of the quality of the included studies was based on the mCMS values as follows: 0–25 (poor), 26–50 (fair), 51–75 (good) and 76–90 (excellent).

### Statistical analysis

Because of the very low number of randomized controlled trials (RCT), the observed high methodological heterogeneity observed across the studies (RCT and non-RCT) and missing data statistically pooling of the data seemed not plausible. Therefore, meta-analysis of the individual study results was omitted.

## Results

A total of 415 records were identified through this database search. One additional record was found by screening suitable articles’ references. Therefore, 416 records were investigated in total. After removing duplicates, 302 records’ abstracts and titles were screened and 274 of them excluded based on the a priori set exclusion criteria. The remaining 28 articles (26 full-text articles and two abstracts) were assessed for eligibility. Another 16 articles were excluded after full-text assessment. Reasons for exclusion were: no injection reported at all (6) [2, 8, 22, 36, 48, 66], no injection into the patellar tendon (8) [12, 21, 25, 26, 54, 56, 59, 70], no new study data (respectively they reported on the same study data of an included study) [60] or animal studies (1) [20]. Thus, twelve articles were included in the systematic review and were suitable for qualitative synthesis. Regarding the abstracts, no full-text article was available even after contacting the corresponding authors [47, 62] Therefore, these studies were also excluded, reducing the final number of studies for further analysis in this systematic review to ten.

Figure 1 shows the detailed selection process in a flowchart according to the PRISMA guidelines. Regarding the type of therapy, eight studies investigating the effect of ST and two studies on PT were included. Referring to the study design, three randomized controlled trials comparing verum injection to placebo injection [31], arthroscopic shaving [67] or placebo injection and usual care consisting of stretching and exercise [64], three prospective studies [3, 33, 53] and one case report [22] were included. In addition, three follow-up investigations of the two ST RCTs [30, 32, 61] were also included, although no new injection was administered, since their findings might provide important information on long-term outcome. The follow-up studies were treated as follow-up of the original study, not as a separate study.

Tables 1 and 2 illustrate the main findings of the included studies. Taken all studies into account, 343 tendons are summarized, 231 (67.3%) of which received ST while 112 (32.7%) tendons were targeted by means of PT. The mean number of injections for patients treated with ST was 2.7. One PT study reported the median number of injections received (median = 4 (range 2–8)) [53] and one study reported a mean number of injections of 3.8 [64]. Gender distribution, when reported across studies was not equivocal (male patients = 174 (90.2%); female patients = 19 (9.8%)). Mean age of the patients was 25.9 years (range 9–58). The mean duration of CPT symptoms, when reported, was 22.4 months and the mean follow-up ranged from 4 to 46 months.

**Table 1:** Demographics

Author	Year	Study type	Patients		No of tendons	Mean age	Mean duration of symptoms	Mean follow-up
			m	f		years	months	months
Sclerotherapy								
Alfredson	2004	prospective	12	3	15	29	23	6
Gisslén	2006	case report	1	0	1	25	9	4
Hoksrud	2006	RCT	28	5	43	24.9	37.1	27
Willberg	2011	RCT	43	2	52	26	22	46
Hoksrud	2012	prospective	101		120	27	17	15
Prolotherapy								
Ryan	2011	prospective	39	6	47	38.3	21.8	11
Topol	2011	RCT	51	3	65	13.3	not reported	12

*m* male, *f* female, *RCT* Randomized controlled trial

**Table 2:** Intervention

Author	Year	Study Type	Substance	Control	Mean VAS			Mean no of injections	Serious Adverse Events
					pre	post	FU		
Sclerotherapy									
Alfredson	2004	prospective	polidocanol 5 mg/ml	na	79.7	19		2.7	none
Gisslén	2006	casereport	polidocanol 10 mg/ml	na	90	0	0	1.0	none
Hoksrud	2006	RCT	polidocanol 10 mg/ml	adrenaline 5 μg/ml+lidocaine hcl 5 mg/ml	VISA	VISA	VISA	3.1 (intervention + crossover)	none
Willberg	2011	RCT	polidocanol 10 mg/ml	arthroscopic shaving	69	41.1	(17)	na	none
Hoksrud	2012	prospective	polidocanol 10 mg/ml	na	VISA	VISA	VISA	2.5	none
Prolotherapy									
Ryan	2011	prospective	25% dextrose solution	na	51.1		25.8	median 4	none
Topol	2011	RCT	12.5% dextrose solution	lidocaine 10 mg/ml	NPPS	NPPS	NPPS	3.8	none

*VAS* Visual Analogue Scale, *no* number, *FU* Follow-up, *RCT* Randomized controlled trial, *na* not available, *VISA* Victorian Institute of Sport Assessment, *NPPS* Nirschl Pain Phase Scale

All ST studies used Polidocanol as sclerosing agent. One study injected Polidocanol in a concentration of 5 mg/ml [3], all other studies applied a concentration of 10 mg/ml [22, 31, 33, 67]. In one RCT the investigators injected a placebo solution made of adrenaline 5 μg/ml and lidocaine 5 mg/ml to obtain the same immediate effect [31]. The PT RCT used 12.5% dextrose solution and lidocaine 10 mg/ml as a placebo solution [64]. The non RCT PT study also used a dextrose solution to treat the patellar tendon. Ryan et al. injected a 25% dextrose solution [53].

### Quality assessment and risk of bias

Mean modified Coleman Score of all studies was 64,57 which is rated as good quality. There was one article defined as fair quality [22], five articles having good quality [3, 33, 53, 64, 67] and one article rated as excellent quality [31]. No study was defined as having poor quality. Table 3 presents an overview of the mCMS assessment.

**Table 3:** modified Coleman Methodology Score

Author	Coleman Score	Rating
*Alfredson 2004*	53	good
*Gisslén 2006*	43	fair
*Hoksrud 2006*	83	excellent
*Topol 2011*	68	good
*Willberg 2011*	73	good
*Hoksrud 2012*	72	good
*Ryan 2011*	60	good
Overall	64.57	good

**Fig. 2:**
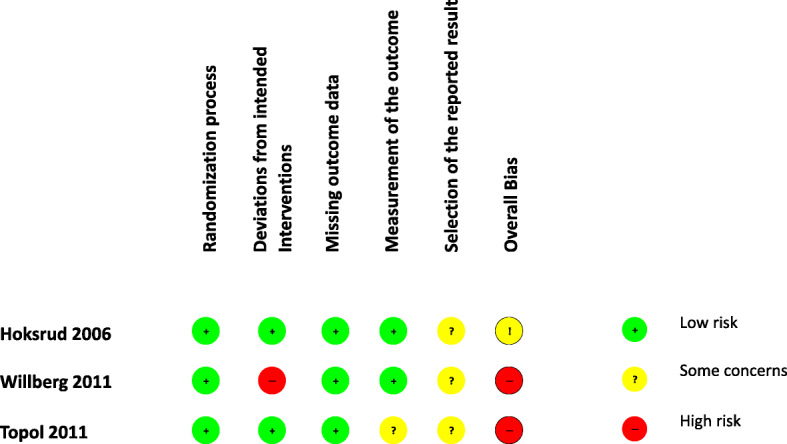
Summary of the risk of bias assessment

Figure 2 shows an overview of the risk of bias (RoB) assessment. The Hoksrud’s RCT had an overall risk of bias rated as “some concerns” because no raw data was available. The Willberg study showed an overall high risk of bias. Since a blinding of the two investigated interventions (shaving vs. injection) was not possible due to obvious reasons, we rated this domain of high risk of bias, which mainly attributes to the overall risk of bias. The Topol study showed also an overall high risk of bias, since the investigators and participants were unblinded after 3 months and further injections were performed. As stated above the application of Cochrane’s RoB Assessment Tool is not developed for non-RCTs. All non-RCTs were treated as having an overall high risk of bias.

### Individual studies

#### RCT Sclerotherapy

Hoksrud et al. compared the effect of Polidocanol against placebo injections containing adrenaline and lidocaine in their double blinded RCT of 2006 [31]. They reported a significant pain reduction, improvement in VISA score and patients’ satisfaction with the treatment. The treatment group showed an increase in VISA-P score from 54 to 62 at the 4 months follow-up, whereas the control group showed no changes in VISA-P score. After 4 months the control group crossed over and received sclerosing injections as well. After 8 months, the VISA-P score for both groups after receiving injections was 75, which also reflects the minimum clinically important difference (MCID). The MCID is defined as an absolute change of more than 13 points, respectively a relative change of 15.4–27% of the baseline VISA-P score [27]. Nearly one third (14 tendons) of the patients received additional treatment (6 patients tendon surgery, 8 patients non tendon surgery (e.g. debridement of chondral defects or resection or removal of a plica medialis) after sclerosing injections. The VISA-P score increased further from 75 (8 months follow-up) to 89 (44 months follow-up) for the patients (23 tendons) who did not receive additional treatment (surgery). For the patients who had undergone surgery VISA-P score improved to 91 (tendon surgery) and 92 (non-tendon surgery) [30]. Twenty-eight of 33 patients were back to full training with no or only mild symptoms after 12 months [31].

In one follow-up investigation, focussing on the ultrasound findings of the study population, the investigators stated, that in about two third of patients with patellar tendinopathy neovascularisations can be expected. They were not able to show a correlation between an improvement in VISA-P score and structural changes after the injection therapy [32].

The second RCT by Willberg and colleagues, investigated the effect of sclerosing Polidocanol injections versus arthroscopic shaving [67]. The authors showed good results for both cohorts but stated that arthroscopic shaving led to a greater pain reduction and more satisfaction with the treatment. The VAS score for the sclerosing group decreased from 69.0 pre-treatment to 41.1 at follow-up. VAS score for the arthroscopy group fell from 76.5 to 12.8 and was significantly lower than VAS for the ST group at follow-up (*p* = 0.001).

In a medium-term follow-up study the same researchers sought to investigate the effect of both treatments after three to 5 years [61]. They reported good clinical outcomes for both treatment modalities, showing a significantly faster rehabilitation for the arthroscopic group. VAS score in the sclerosing group further decreased to 17 but remained stable (13) in the arthroscopic group. There was no statistically significant difference between both groups. For both groups, the researchers found significant changes referring to structural changes and neovascularisation in the ultrasound examination (ST group *p* = 0.013, arthroscopy group *p* < 0.001). Furthermore, positive and statistically significant relationships between the existence of neovascularisations and VAS score during activity (r = 0.63, *p* < 0.0001) and between the VAS score and structural changes (r = 0.52, *p* < 0.0001) were observed.

#### RCT Prolotherapy

Topol et al. investigated the effect of hyperosmolar dextrose injections to treat Osgood-Schlatter Disease (OSD) in children and adolescents [64]. They divided 54 patients with clinically diagnosed OSD in 3 groups. The control group had to do 3 months of supervised stretching and exercise therapy, the placebo group received monthly injections of lidocaine 10 mg/ml and the intervention group was treated with 12.5% hyperosmolar dextrose solution. At the 3 months follow-up the intervention group showed a bigger drop in Nirschl Pain Phase Scale (NPPS) compared to the other groups. Mean NPPS difference after 3 months was 3.9 for the intervention group compared to 2.4 for the placebo group and 1.2 for the control group (*p* < 0.0001 between all groups). After 3 months investigators and patients were unblinded and all groups offered further monthly dextrose injections for at max 9 more months until a satisfying level of symptom control was achieved. After 12 months they showed a higher success rate in the patients receiving dextrose injections compared to the patients not receiving dextrose injections (32/38 vs. 6/13, *p* = 0.024). All patients of the intervention group could participate in sports after treatment and 14 out of 21 knees were asymptomatic [64].

#### Non-RCT Sclerotherapy

This search led to three non-RCT articles treating the patellar tendinopathy with sclerosing injections. Alfredson et al. carried out a pilot study with a prospective design in 2004. Fifteen patients with sonographically confirmed diagnosis of patellar tendinopathy received a mean of 2.7 injections (SD = 1.58) of Polidocanol (5 mg/ml). The mean baseline VAS was 79.7 and reduced to 19 (*p* < 0,001) in the follow-up examination. Twelve out of 15 patients were satisfied with the treatment. Beside the VAS, the amount of neovascularisation was documented with a score ranging from zero to four. The mean baseline neovascularisation was estimated 3.87 (SD = 0.35) and decreased to 1.69 (SD = 1.54, *p* < 0.001) after sclerosing treatment [3].

In 2012 the research group of Hoksrud conducted a prospective trial with 120 tendons (101 patients) [33]. The researchers injected Polidocanol in a concentration of 10 mg/ml from the ventral site of the tendon in a mean of 2.5 injections. Twenty-two patients had additional knee surgery during the follow-up period and additional 29 patients underwent non-surgical treatment options. Hoksrud et al. reported a moderate improvement in VISA-P score. For all patients who were followed up, VISA-P increased from 39 at baseline to 65 at the 24 months follow-up. The group that was treated without surgery showed an increase in VISA score from 39 to 68, while the surgery group had an increase from 43 to 57.

In a case study, Gisslén et al. cured a 25-year-old Olympic weightlifter suffering from CPT. After one injection of Polidocanol (10 mg/ml) and a two-week rehabilitation phase, the athlete was back to full tendon load activity. VAS before the treatment during weightlifting was 90 and went down to 0 during weightlifting after ST. Furthermore, Gisslén et al. reported on a reduction of neovascularisation in the 4 months follow-up [22].

#### Non-RCT Prolotherapy

One study on PT could be included in this systematic review. Ryan et al. investigated in a prospective pilot study the effect of a 25% dextrose solution to treat the painful patellar tendon. The mean baseline VAS in daily living for the 47 included patients was 51.1 (SD = 22.9). After having received a median of 4 injections (±3), VAS significantly fell to 25.8 (SD = 20.1) (*p* < 0.001). More than half of all patients (25 out of 47) stated reduction of pain of more than 50%. Furthermore, the authors implied that dextrose injections might enhance tendon remodelling, since they could show a decrease in tendons with intratendinous tears (22 to 10 tendons) and reduction of hypoechogenicity (13 to 5). Nevertheless, Ryan et al. could not show a relationship between neovessels and tendon pain [53].

### Adverse effects

All trials stated that there occurred no serious AE (SAE) during the treatment. However, in the Ryan study, three patients reported an increase in pain after treatment, which can be classified as AE [53]. In the Topol study < 10% of the participants made use of acetaminophen to control postinjection pain [64].

## Discussion

The aim of this systematic review was to evaluate the effect of sclerotherapy (ST) or prolotherapy (PT) on pain and function in patients suffering from CPT. By systematically searching electronic databases and references all available studies on this topic should be included to be able to make a clear statement on the effectiveness of both therapies.

The two RCTs on ST showed positive and clinically relevant results in terms of a reduction in pain or an increase in function, respectively. However, in the Willberg study, pain was statistically significant lower in the arthroscopy group as compared to the sclerotherapy group indicating a better improvement in pain scores after arthroscopy.

The RCT on PT confirmed the findings with all patients of the intervention group participating in sports and 14 out of 21 asymptomatic knees. At first sight OSD might not appear as a typical tendinopathy condition, but OSD ultrasound studies suggest that tendinopathy features including neovessels can be found in the distal patellar tendon [16, 39, 55]. Therefore, this study was included.

The non-RCT studies on ST and PT underlined the positive findings of the RCTs. Only one study reported on a moderate improvement. This might be attributed to the fact, that the injection was performed from the ventral side of the patellar tendon. It is recommended to perform the injection from the dorsal side of the patellar tendon where the neovessels enter the tendon [3, 17].

The methodological quality assessment of the included studies demonstrated good quality (mCMS = 64.57 points). Nevertheless, there was only one of the included studies rated with excellent methodological quality. This clearly reveals the need of more high-quality studies. Methodological quality was flawed by no adequate randomization and lack of control group. No study was of poor methodologic quality.

Risk of bias assessment showed an overall high risk in two RCT study. In one of these studies, a blinding of the two interventions was not feasible, which resulted in a high risk of bias. In the other study, patients and physician were unblinded after 3 months. We defined all non-RCTs as high overall risk of bias, which underlines the need for further RCTs. All RCT were rated of “some concerns” in the domain “selection of the reported results” because we had no raw data available. All other domains were rated of low quality. Hence, there conclusion and results remain usable within the constraints of very high likelihood for risk of bias.

To our knowledge this is the first systematic review focussing only on the effectiveness of ST and PT on pain and function in patients with CPT. Therefore, it is difficult to compare it to similar reviews. Larsson et al. conducted a systematic review of RCTs on treatment options in patients with patellar tendinopathy. Their main outcome was the VISA-P score, respectively VAS score. They included 13 studies with 612 patients in total. Larrson et al. stated that eccentric squats have the best evidence, whereas ST among others might have a benefit as well. They were not able to make a clear statement, since they included only one study on sclerotherapy [40]. Two more recent systematic reviews on the treatment of CPT concluded also that that eccentric training has the best evidence while there was limited evidence for ST [19, 40] to have an effect on pain and function in patients with CPT. Among other factors this is accounted for by the lack of more suitable studies. What all these reviews have in common is the statement, that more high-quality research and studies are needed to be able to make recommendations for the use of ST and PT in clinical routine when working with patients suffering from CPT.

Since the selected studies were found to be unsuitable for a quantitative synthesis and no meta-analysis was conducted, a clear statement on the clinical relevance of an overall effect size of ST and PT on pain and function in patients with CPT cannot be presented. Yet, based on the limited set of studies included in this analysis and taking in considerations the high likelihood for risk of bias in the selected studies, the qualitative synthesis of the individual studies’ effect sizes suggests a promising clinical effect. Nevertheless, the true size of this effect is still to be determined, which underlines the need of more research in this field.

### Safety

Regarding safety of the interventions, PT and ST can be considered safe, as three AEs (increase in pain), but no serious adverse events (SAE) were reported. Taken all included studies, there were multiple injections performed in 343 tendons. As far as we know, there is no existing definition of a safe intervention regarding the occurrence of AEs. Bearing in mind that the total number of injections and tendons is small for a final conclusion and most studies are underpowered to detect AEs, since they did not comment on a systematic AE screening, we are of the opinion that both therapies can be considered safe. A recent systematic review on the effect of ST and PT on painful Achilles tendinopathy underlined these findings. The authors reported on only three, partially debatable, adverse events compared to more than 600 injections [46]. The adverse events occurring were a lesion of Nervus suralis due to an injection from the lateral side, although the injection is normally performed from the medial side to reduce the risk of lesions, one case of Embolia cutis medicamentosa, and a cutaneous reaction after injection, which can lead to necrosis and a questionable partial tear of the Achilles tendon [13, 35, 44]. In their well-designed systematic review, Coombes et al. sought to investigate the efficacy and safety of several injection therapies on tendinopathy. They acknowledge that beside pain while injecting, sclerosant and prolotherapy injections are a safe procedure [15].

As case numbers are small for treating tendinopathy, the safety of both procedures still need to be investigated and should be addressed in further trials. General indications of possible complications using Polidocanol or any sclerosing agent can come from its origin – sclerosing varicose veins. The treatment is known to be safe, and complications are very rare. A big multicentre registry study observed in 12,173 sessions 49 (0.4%) complications [23]. The Australian Polidocanol study came to the same conclusion. In 16,804 injections into varicose veins, spider veins and venules, there were 154 (0.9%) complications (including allergic reactions) [14]. As mentioned before this has to be understood as a general indication since there were no injections in patellar tendons. It seems to have foamed sclerosants connected to more adverse events than liquid sclerosants (0.22% vs. 0.58%) [23]. Even serious neurological events are described for patients with patent foramen ovale after using foamed sclerosant [11, 38].

### Limitations

This systematic review, aimed to provide an extensive overview on the effectiveness of ST and PT on pain and function in patients with CPT, has some limitations. First, there is some potential risk of incomplete retrieval bias. Although a comprehensive search strategy was developed, it cannot be assured to really have captured all existing trials on this topic. Having included all types of study designs made the search more sensitive and may have reduced the methodological quality of the included studies to a certain extent. Unfortunately, based on this small set of retrieved studies, it was not plausible to perform a meta-analysis since between studies’ heterogeneity was too high and raw data of three out of the ten studies were missing. A quantitative synthesis could have assessed a more accurate possible clinical effect size of ST and PT procedures on pain and function in such patients. At the same time this emphasizes the demand of more methodological well-performed studies. Most of the studies were set up in a single-arm design. Thus, there is a potential risk of bias, since we cannot exclusively assign the positive effects to the injections. Unfortunately, some authors did not respond to our request on raw data and additional information on their studies. This prevents us from being able to draw more conclusive statements. Regarding data extraction, we defined the three-month follow-up as main follow-up point. Unfortunately, follow-up time points were not consistent. Therefore, we extracted all available follow-ups. As a result, comparability of follow-ups is reduced. Furthermore, we have to note the missing comparison to other treatments. As the Willberg study showed a greater pain reduction and satisfaction with the arthroscopic shaving, it would be of interest how sclerotherapy and prolotherapy perform compared to other treatments. As the ESSKA emphasizes, the most common approach to treat patellar tendinopathy is an exercise-based treatment [1]. To strengthen the recommendation for the use of sclerotherapy and prolotherapy a RCT with sclerotherapy compared to an exercise-based regime could have a big impact.

Some questions are still unanswered and should be addressed in upcoming studies. It is still debatable, whether ST or PT is favored when comparing their effect on pain and function in patients with CPT. Thus, more direct head-to-head high-quality trials are needed. Furthermore, in such studies important covariates should be analyzed. For example, it is still not clear, in which dose and concentration Polidocanol is used most effectively to reduce pain and increase function in patients with CPT. Most of the studies used up to 2 ml per injection of 10 mg/ml Polidocanol resulting in a mean of 2.7 injections per patient to treat the patellar tendinopathy. Comparing these findings to the systematic review on Achilles tendinopathy, where injections of 5 mg/ml Polidocanol resulted in a mean of 2.6 injections, it is questionable whether the concentration makes a decisive difference. A RCT from Willberg comparing two different concentrations of Polidocanol can confirm this consideration [68].

It is noteworthy to mention that PT shows a mean of approximately 4 injections. Most of the studies had an interval of 4 weeks, resulting in a treatment duration of 16 weeks for the PT treatment. Comparing both therapies under this point of view, one can assume ST being superior, since treatment duration will be shorter. This might have a significant impact on patients, especially on athletes during competition season. Following this consideration, the question of shortening the interval remains unanswered as well. Having a short duration of treatment could make the injection treatments more attractive to athletes, since their return to sport and competition will be sooner. In comparison the standard procedure of eccentric training provides at least a treatment period over 12 weeks and is known to be painful [37]. Therefore, future studies should focus on shortening the interval.

Further it is conceivable that a combination of different treatment options can improve the outcome. Yelland et al. showed a faster improvement in VISA-A score combining Prolotherapy and eccentric training [69]. In another recent study a combination of eccentric training and high-volume injections with and without corticosteroids demonstrated promising results [9]. This suggests further research on combined treatment schemes.

## Conclusions

Based on the limited set of studies included in this systematic review it seems that there is but very weak evidence that ST and PT might be effective treatment options to reduce pain and increase function in patients with CPT. The present review also showed that 3 out of 295 injections (about 1%) caused even more pain. This finding is corroborated with other studies and this number of AEs is considered to be acceptable. Due to a lack of high-quality RCTs, a strong recommendation for daily use in the clinical setting when treating such patients cannot be made to date. This emphasizes the need of more high-quality RCTs in a double-blinded design and including direct head-to-head comparisons to underline the effectiveness and use of ST and PT. Finally, once effectiveness of the treatment alternatives has been established, their financial efficiency should also be analyzed using cost-effectiveness studies or cost-utility studies.

## Supplementary Information

**Additional file 1.** The Ovid Search Strategy.

## Abbreviations

AE: Adverse Event
AT: Achilles Tendinopathy
CPT: Chronic Patellar Tendinopathy
FU: Follow-Up
MCID: Minimum Clinically Important Difference
mCMS: Modified Coleman Methodology Score
NPPS: Nirschl Pain Phase Scale
OSD: Osgood-Schlatter Disease
PICOS: Population, Intervention, Outcome, Study design
PRISMA: Preferred Reporting Items for Systematic Reviews and Meta-Analyses
PT: Prolotherapy
RCT: Randomized-Controlled Trial
RoB: Risk of Bias
SAE: Serious Adverse Event
SD: Standard Deviation
ST: Sclerotherapy
VAS: Visual Analogue Scale
VISA-P score: Victorian Institute of Sport Assessment - Patellar score
