# Supplementary material for: Sclerotherapy and prolotherapy for chronic patellar tendinopathies - a promising therapy with limited available evidence, a systematic review
Source: J Exp Orthop. 2020 Nov 9;7:89. doi: 10.1186/s40634-020-00303-0 (PMC7652964; doi:10.1186/s40634-020-00303-0)
Supplement: Supplementary file 1 — Additional file 1. The Ovid Search Strategy. [file 40634_2020_303_MOESM1_ESM.pdf]

APPENDIX
The Ovid Search Strategy

1. (Pol#do#anol or POL or SCH-600 or Asclera or aethoxys#lerol or ethoxys#lerol or Varithena or Varisolve or polidocanol endovenous microfoam or foam sclerosant or Dodecylethanol or laureth or aetoxisclerol or atossisclerol or atoxysclerol or Dodecylheptaglycol or Dodecylnonaglycol or Lauromacrogol or Lauro-macrogol or macrogol or PEG-9 lauryl alcohol or Sodium tetradecyl sulfat* or STS or Sotrade#ol or Tergitol or Trombavar or Trombovar or Scleromate or Morrhuate Sodium or Morr?uate or Varicoccid or sclerosing agen* or sclerosing solu* or sclerosing subs* or polyethylen* or Ethanolamin* oleat* or Et?amolin or Monoet?anolamin* oleat* or Oldamin or Et?amsylat* or Altodor or Cyclonamin* or Dic#none or Dic#nene or Hemo 141 or Hemo141 or alcoholic prolamine solution or Zein or Et?ibloc or Ok?432 or Picibanil).mp.
2. Sodium Tetradecyl Sulfate/
3. Sclerosing Solutions/
4. Polyethylene Glycols/
5. Polidocanol/
6. Ethamsylate/
7. 1 or 2 or 3 or 4 or 5 or 6
8. Glucose/ad, ae, pd, tu, th, to [Administration & Dosage, Adverse Effects, Pharmacology, Therapeutic Use, Therapy, Toxicity]
9. Glucose Solution, Hypertonic/
10. Phenol/
11. Tannins/
12. Guaiacol/
13. Glycerol/
14. Zinc Sulfate/
15. ((hypertonic adj5 dextrose) or (hypertonic adj5 glucose) or phenol-glucose-glycerine or p2g or gluco* or dextro* or proliferat* or tannic acid or guaiacol or guaiakol or phenol or glycerin* or zinc sulfat*).mp.
16. ((gluco* or dextro*) and hyperosmolar).mp.
17. 8 or 9 or 10 or 11 or 12 or 13 or 14 or 15 or 16
18. 7 or 17
19. Injections/
20. Injections, Intralesional/
21. (inject* or (intralesion* adj5 inject*) or drug administra* or infiltra*).mp.
22. ("intra-lesional" and "inject*").mp.
23. 19 or 20 or 21 or 22
24. Sclerotherapy/
25. Prolotherapy/
26. (sclerosing* or sclerothera* or sclero-thera* or prolothera* or prolo-thera* or (proliferation adj5 thera*) or conservative* or (regenerative adj3 inject*)).mp.
27. 24 or 25 or 26
28. 23 or 27
29. 18 and 28

30. Patellar Ligament/
31. (patel?a* tendon* or patel?a* ligament*).mp.
32. 30 or 31
33. Tendinopathy/
34. Tendon Injuries/
35. Athletic Injuries/
36. Soft Tissue Injuries/
37. Knee Injuries/
38. Cumulative Trauma Disorders/
39. Musculoskeletal Diseases/dt, et, pa, rh, th [Drug Therapy, Etiology, Pathology, Rehabilitation, Therapy]
40. Tenosynovitis/
41. tendinopath*.mp.
42. athletic injur*.mp.
43. sports injur*.mp.
44. soft tissue injur*.mp.
45. overuse synd*.mp.
46. (cumulative adj5 trauma disorder*).mp.
47. overuse injur*.mp.
48. overexertion injur*.mp.
49. tendin*.mp.
50. tendon*.mp.
51. teno*.mp.
52. paratend#nitis.mp.
53. peritend#nitis.mp.
54. ent?esopath*.mp.
55. jumper* knee.mp.
56. 33 or 34 or 35 or 36 or 37 or 38 or 39 or 40 or 41 or 42 or 43 or 44 or 45 or 46 or 47 or 48 or 49 or 50 or 51 or 52 or 53 or 54 or 55
57. 32 and 56
58. 29 and 57
